# A longitudinal study of socioeconomic status, family processes, and child adjustment from preschool until early elementary school: the role of social competence

**DOI:** 10.1186/s13034-017-0206-z

**Published:** 2017-12-19

**Authors:** Rikuya Hosokawa, Toshiki Katsura

**Affiliations:** 1 School of Nursing, Nagoya City University, Mizuho-cho, Mizuho-ku, Nagoya, 467-8601 Japan; 2 Graduate School of Medicine, Kyoto University, Kyoto, Japan

**Keywords:** Socioeconomic status, Marital conflict, Parenting practice, Social competence, Behavioral problems, Preschool children

## Abstract

**Objective:**

Using a short-term longitudinal design, this study examined the concurrent and longitudinal relationships among familial socioeconomic status (SES; i.e., family income and maternal and paternal education levels), marital conflict (i.e., constructive and destructive marital conflict), parenting practices (i.e., positive and negative parenting practices), child social competence (i.e., social skills), and child behavioral adjustment (i.e., internalizing and externalizing problems) in a comprehensive model.

**Methods:**

The sample included a total of 1604 preschoolers aged 5 years at Time 1 and first graders aged 6 years at Time 2 (51.5% male). Parents completed a self-reported questionnaire regarding their SES, marital conflict, parenting practices, and their children’s behavioral adjustment. Teachers also evaluated the children’s social competence.

**Results:**

The path analysis results revealed that Time 1 family income and maternal and paternal education levels were respectively related to Time 1 social skills and Time 2 internalizing and externalizing problems, both directly and indirectly, through their influence on destructive and constructive marital conflict, as well as negative and positive parenting practices. Notably, after controlling for Time 1 behavioral problems as mediating mechanisms in the link between family factors (i.e., SES, marital conflict, and parenting practices) and behavioral adjustment, Time 1 social skills significantly and inversely influenced both the internalization and externalization of problems at Time 2.

**Conclusions:**

The merit of examining SES, marital conflict, and parenting practices as multidimensional constructs is discussed in relation to an understanding of processes and pathways within families that affect child mental health functioning. The results suggest social competence, which is influenced by the multidimensional constructs of family factors, may prove protective in reducing the risk of child maladjustment, especially for children who are socioeconomically disadvantaged.

## Background

An extensive amount of research has consistently found associations between childhood socioeconomic status (SES) and mental health functioning [1–3], with marital conflict and parenting practices seeming to mediate these associations. SES is a construct that consists of multiple dimensions of social position [4, 5]. Previous related empirical and theoretical research has focused on economic and educational aspects as SES indicators. Family income has been associated with children’s developmental outcomes, as have parental educational levels [6–12]. However, despite the many studies conducted in this area, few have simultaneously investigated the influence of family income and maternal and paternal education levels as predictors in the relationships between SES, family processes (e.g., marital conflict and parenting practices), and child mental health functioning.

Additionally, despite extensive studies concerning the relationships between SES, family processes, and child mental health functioning, most have only minimally considered the effects of the positive dimensions of marital conflict and parenting practices (e.g., constructive marital conflict and positive parenting practices), rather than the negative dimensions thereof (e.g., destructive marital conflict and negative parenting practices), as mediators in the link between SES and child mental health functioning [7, 13–16]. Moreover, a limitation of previous empirical work concerning these associations (i.e., SES, family processes, and child mental health functioning) is that these studies focused on negative developmental outcomes (e.g., internalizing and externalizing problems) [17, 18]. Further studies examining positive dimensions of child mental health functioning, especially the issue of social competence, are needed. Social competence, which is defined as an individual’s ability to act in a socially appropriate manner [19, 20], has received comparatively less attention as a mediator in the link between SES, family processes, and child behavioral adjustment, despite preliminary evidence suggesting it may be an important indicator.

When considering the complex relationships between these variables, it is important to consider independent associations, while controlling for other variables. However, previous studies have primarily examined individual relationships between different types of SES, marital conflict, and parenting practices, as well as child social competence and behavioral adjustment, without considering these associations in a comprehensive model. Therefore, this study examined mediators of the associations between SES and children’s functioning in greater detail. Specifically, destructive and constructive marital conflict, negative and positive parenting practices, and child social skills were investigated as mediators in the associations between SES indicators, including family income and parental education levels, and children’s internalizing and externalizing behaviors in a unified model. Regarding social skills, we especially focused on the mediating role of social competence in the relationships between family factors (i.e., SES, marital conflict, and parenting attitude) and child behavioral problems, from preschool to the first grade.

### Socioeconomic status and child adjustment

Research in the past decade has shown that SES is an important contextual factor that strongly predicts child outcomes [1–3]. Extensive research has shown that SES affects the well-being and development of children, including their internalizing (e.g., anxiety, depression, and withdrawal) and externalizing (e.g., aggression, opposition, and hyperactivity) symptoms, as well as their cognitive and language development [1, 3, 21–27].

It has been well documented that economic problems, such as low income and financial instability, adversely influence inter-parental and parent/child interactions, which in turn are related to a range of harmful outcomes for child development [28]. Studies have shown that economic problems are associated with destructive parental interactions that predict increased domestic problems and lower levels of marital quality. Furthermore, it has also been shown that economic problems place children at an increased risk of exposure to family conflict [7, 29–32]. Economic problems are also predictors of negative parenting, including lack of warmth and involvement, parental harshness, and authoritarian parenting methods [28, 33–36].

The family stress model (FSM), which was proposed by Conger et al., explains the relationships among SES, marital conflict, and parenting style, while also providing solid evidence for the negative effects of family economic problems on both parents and children [15, 37]. The FSM proposes that economic hardship predicts economic pressure, which in turn exacerbates emotional distress (e.g., depression, anxiety, anger, and alienation) for both parents [37]. In turn, parental emotional distress has a direct, negative impact on the parents’ relationships with each other, as indicated by conflict. This conflict then spills over into parent/child relationships, in the form of negative parenting, resulting in harsh, uninvolved, and/or inconsistent child-rearing practices; these parenting styles are associated with an increase in negative outcomes for children [29, 37–39].

Educational status and economic aspects are typical quantitative SES indicators [4, 5]. Many previous studies have focused on the educational aspects of SES in the relationship between SES and child development, with parental educational levels being associated with child developmental outcomes [1, 2, 10–12, 25, 26]. However, despite the many studies completed in this area, few have simultaneously investigated the influence of multiple components of SES, including family income, and maternal and paternal education levels, as predictors in the relationships among SES, family processes, and child mental health functioning. In several studies that include both educational and economic aspects of SES indicators, educational status has often either previously been used as a control variable, or it has been combined with income in the construction of an overall index of SES indicators [6, 7]. Furthermore, a limitation of previous empirical work on the FSM is that studies have also focused exclusively on the economic aspect of SES in the relationship between SES and family processes, dedicating little research attention to the educational aspects of SES [28]. It is well known that education is an important predictor of family income across the life course [40]. Therefore, it may be reasonable to expect the influence of educational status on parental interactions and parent/child interactions to be indirect and mediated by economic well-being.

Education is an important component of SES that helps identify a social class or position, and has been linked to individual competence [4]. Higher education is likely to enhance various individual skills for competent functioning, such as problem-solving skills, cognitive skills, and capacity to cope with change. People with higher levels of education tend to be able to solve problems that are more complex and perform jobs with more autonomy and creativity [41–44]. Moreover, educational achievement provides persons with more employment opportunities, enhances their ability to make significant contributions to their fields, and demonstrates significant positive associations with occupational prestige and income [40, 45–47]. Furthermore, according to human capital theory, the education level of an individual’s spouse also helps accumulate human capital and has an important impact on economic outcomes [48, 49]. For example, a spouse with a higher education might provide constructive advice and information that can affect career and decision making in the family, such as consumption, fertility, and where to live [50–52]. Additionally, spouses are likely to affect each other through values, attitudes, and other abilities associated with education. Many studies have revealed common findings that the education level of an individual’s spouse is positively correlated with the individual’s earnings. Especially, numerous studies have suggested that a wife’s education affects her husband’s earnings [51–56], and vice versa. Additionally, other studies have shown that an individual’s earnings are positively correlated with their spouse’s education level [53, 57]. This correlation might be due to marital matching, as individuals that are more productive are more likely to marry better-educated individuals.

However, despite the fact that parental education levels strongly interact with income, education levels and economic conditions could have different effects on family processes and child mental health functioning, possibly acting through different pathways. Regarding the relationship between educational level and marital relationship, higher education is likely to help parents to strengthen their communication and analytical skills, allowing for more effective problem solving between parents [44, 50, 58]. Moreover, higher education is also likely to enhance self-control and coping mechanisms of parents, possibly increasing the positive association between education and psychological well-being [58]. Consequently, parental education levels might positively affect marital relationship through parental psychological well-being [44, 59–61]. A large amount of evidence for the beneficial nature of education on marriage exist, as studies have demonstrated a negative relationship between parental educational levels and marital conflict [62], a positive association between educational attainment and greater marital satisfaction [30, 63], and higher levels of educational attainment are associated with greater marital stability [64, 65].

In addition, previous research has suggested that parental education is the strongest and most important predictor of parenting behavior [66]. Regarding the relationship between educational level and parent/child interactions, higher education is likely to promote the ability to process information, and enable parents to acquire more knowledge and skills about childrearing and child development, allowing parents with higher education to use more effective strategies for childrearing [66–68]. Moreover, as mentioned above, a higher level of education is likely to boost parental psychological well-being, which, in turn, could positively influence parenting style [69–71]. Many studies found that higher maternal education levels are associated with more supportive parenting [72, 73], which is also associated with positive cognitive, behavioral, emotional, and physical child outcomes [74–77]. While few studies have investigated the influence of paternal education levels on fathers’ involvement in childrearing, some studies have found paternal education levels to be somewhat associated with parent/child interactions. For example, several studies revealed that fathers with higher educational attainment tend to be more involved, show more positive engagement, and be more accessible to their children than fathers with a lower education level [78–80]. However, other studies have found little association between paternal educational attainment and fathers’ involvement, after controlling for factors such as family income and maternal education level [6–9]. As there are conflicting results in the literature regarding the influence of paternal education level on parental involvement, it is possible that parental education levels may influence parenting attitudes directly, or they may do so indirectly through family economic factors or other SES indicators. Given this information, we are unable to form strong expectations regarding the possible pathways of how both maternal and paternal education levels may influence childhood mental health problems.

When considering the complex relationships in the above-mentioned variables, it is important to consider independent associations, while controlling for other SES variables. However, few previous studies have primarily examined individual relationships between SES, including family income and parental educational levels, inter-parental interactions, parent/child interactions, and/or child mental health functioning, taking into account associations in a comprehensive model. Therefore, investigations into SES, including family income and parental educational levels, are needed to clarify how each SES indicator flows through the family processes to influence child development. Studying individual markers of SES, including family income and maternal and paternal education, enables us to study the unique and combined contributions of family income and parental education towards family functioning and child adjustment.

### Family processes and child adjustment

As mentioned earlier, the FSM has shown that economic hardship predicts greater economic pressure, in turn exacerbating emotional distress among parents, which then negatively affects their relationship with each other, as indicated by parental relationship conflict [29, 39]. This marital conflict spills over into parent/child relationships, which are characterized by more hostile, harsh, emotionally neglectful parenting, and less warmth. These types of relationships are associated with more negative outcomes (e.g., emotional, behavioral, mental, and physical health problems) in childhood and adulthood [7, 15, 16].

The “spillover hypothesis” has been proposed to explain this relationship between marital conflict and child outcomes. According to this hypothesis, the negativity and positivity experienced in the inter-parental relationship transfer to the parent/child relationship, affecting child outcomes [17, 18, 81–83]. The hypothesis further posits that destructive marital conflict, such as verbal and physical aggression, requires excessive energy that makes parents less emotionally available and less sensitive to the needs of their children. The negative interactions “spill over” into the parent/child relationship, resulting in an increase in negative parenting practices, such as poor monitoring, inconsistency, and harsh discipline. In contrast, constructive marital conflict, such as satisfaction, support, and positive interaction, spills over into the parent/child relationship, which is characterized by increased availability to meet children’s needs, and results in more positive parenting practices, such as involvement and praise. Moreover, several studies examining the effects of conflict on children’s emotional and behavioral outcomes, have also demonstrated ways of categorizing conflict into destructive and constructive marital conflict [84–88]. These studies suggest that destructive marital conflict make children more vulnerable to developing adjustment problems including aggression, conduct disorders, anxiety, and depressive symptomatology. Conversely, these studies also suggest that constructive marital conflict, including progress towards the resolution of the conflicts and explanations about how conflicts were resolved, is likely to be beneficial to children, helping them learn effective problem-solving and communication skills. Therefore, the findings illustrate the need to examine marital conflict as a multidimensional construct to understand how conflict affects children.

However, despite the extensive research completed in this area, studies have minimally considered the impact of positive dimensions of marital conflict and in turn, parenting practices (positive spillover), rather than negative dimensions (negative spillover), as mediators in the link between SES and child mental health functioning. Previous studies have consistently found that destructive marital conflict fosters negative spillover, resulting in more negative parent/child interactions [18]. Furthermore, a limitation of previous empirical work is that studies have focused exclusively on negative outcomes (e.g., internalizing and externalizing behavioral problems) [17, 18]. Further studies examining a positive association between family factors and child mental health functioning, including positive outcomes, have been called for. Therefore, investigations into positive spillover practices (i.e., constructive marital conflict, positive parenting practices, and positive child outcomes) are needed to clarify how family functioning affects child development in a comprehensive model.

### Social competence and child adjustment

School maladjustment is one of the most prevalent and significant health problems threatening children. Previous studies have suggested that one of the factors related to child maladjustment is a child’s inability to adjust socially, as a result of a lack of social competence [89]. Social competence has been broadly defined as effectiveness in social interactions [20]. Social skills are discrete abilities that contribute to social competence [19]. Specifically, these skills have been defined as socially acceptable learned behaviors that enable children to interact effectively and avoid unacceptable responses from others [90]. In short, social competence refers to an individual’s overall ability to act in a socially appropriate manner [19], whereas social skills refer to specific and distinct behaviors representing social competence [91].

Social skills are some of the most important accomplishments in childhood. Aspects of social skills, such as cooperation, self-control, and assertion, which were clustered by Gresham and Elliott [90], affect social adaptation in later life. Social skills help children initiate positive peer interactions, which help them learn positive behaviors through peer modeling and provide them with resources, such as support and acceptance [92–95]. Conversely, children who fail to develop social skills in early developmental phases often display social problems. Children who persistently exhibit deficits in social skills experience both short- and long-term negative consequences, which may often be precursors to more severe social problems later in life [96, 97]. Children who lack social skills may experience emotional difficulties, and tend to have trouble interacting with their peers, teachers, and families [97–100]. Furthermore, social skill deficits frequently demonstrate a negative association with behavioral adjustment [99–102].

Behavioral adjustment is generally associated with two broad symptom dimensions: internalizing and externalizing behaviors. Internalizing behaviors include worry, anxiety, depression, and somatic complaints; while externalizing behaviors include hyperactivity, inattention, aggression toward peers, and management problems [103–110]. Internalizing and externalizing behaviors consistently influence each other over time, with prior studies showing that internalizing behaviors predict later externalizing behaviors, and vice versa [111–116]. Further, there is evidence of co-morbidity with internalizing and externalizing behaviors later in the life course.

Social competence predicts internalizing and externalizing behaviors across longer periods in childhood, adolescence, and adulthood. Additionally, lower social competence forecasts higher levels of both internalizing and externalizing problems [99–102, 117, 118]. Children who lack social skills have difficulties in expressing themselves and understanding others, such as sending appropriate social messages and responding to their peers, teachers, and families. They have fewer positive interactions and have more trouble interacting with others. Consequently, these individuals are more prone to be disliked and deemed socially incompetent by others [119]. Therefore, children with social skill deficits are at an elevated risk for social isolation, including anxious solitude and peer rejection.

Social isolation is associated with behavioral adjustment. For instance, increased childhood social isolation longitudinally predicts depressive symptoms [120–122]. Therefore, early peer difficulties with social skill deficits are predictive of later maladjustment. The cross-sectional and longitudinal associations between social competence deficits and internalizing symptoms have been well documented from preschool to adolescence [123–125]. Similarly, several studies suggest childhood peer rejection longitudinally predicts externalizing behaviors, including aggression, conduct disorders involving peers, and other under-controlled behaviors during the school-age years and into adolescence [101, 102, 126]. However, several social skill abilities among children that are associated with externalizing behaviors, such as abilities in emotion regulation, verbally expressing emotions, and self-regulation of behavior, generally increase with age [127, 128]. Therefore, as social skills improve with age, the rates of externalizing problems tend to decrease in comparison to internalizing problems [127–129]. Eventually, the failure to develop social skills and successful childhood interpersonal relationships could promote mental health difficulties and both internalizing and externalizing problems over time.

Early childhood is a pivotal period for social development. The transition period from early childhood to elementary school first grade is a pivotal period for social development that leads to school readiness. Previous research has indicated that the preschool years are a sensitive period for the acquisition of social skills and related abilities [130–135]. Preschool-aged children learn and frequently display various prosocial behaviors [136]. Therefore, this period is an important developmental stage during when children are expected to acquire social skills to prepare them for broader social activity. Social skill deficits in early childhood gradually become permanent over time, are related to poor academic performance, and are predictive of social adjustment problems and serious psychopathology in adolescence. Understanding the factors that influence these developmental processes in early childhood may enable the prevention of later socio-emotional difficulties.

There is an extensive body of literature demonstrating that the development of social competence among children is significantly affected by environmental factors in childhood [137–139]. For example, family functioning (e.g., the inter-parental relationship, parent/child interactions) has been shown to predict children’s social competence. Positive parenting, such as emotional expressiveness, responsiveness, and support, has been shown to enhance empathy and social functioning in children [140–143], while negative parenting behavior, such as harsh discipline, emotional neglect, or rejecting behavior, is often associated with lower sociability/social competence and increased problem behaviors in children [16, 25, 143].

Many previous studies have also shown that destructive marital conflicts negatively affect social competence [144]. This type of marital conflict may put children at risk of developing adjustment problems, including internalizing and externalizing disorders, due to their inability to control their emotions. Moreover, they may learn through these interactions to solve problems through aggressive behavior [18, 145–147]. Since research has primarily focused on destructive marital conflict, few studies have investigated constructive marital conflict, which may foster social competence. Constructive marital conflict may also aid in the development of problem-solving, coping, and conflict resolution abilities by teaching children how to effectively communicate with others to solve issues [148–150]. Previous studies consistently suggest that destructive conflict increases the risk of adjustment disorders, whereas constructive conflict may positively influence adjustment. Despite the differential effects of destructive and constructive conflict on child development, there is no distinction between these two types of conflict and their implications for social development within the literature. Moreover, even though marital conflict and parenting practices affect social competence [144, 151], few studies have addressed the various ways that this may occur within a comprehensive model.

As mentioned previously, a limitation of empirical work on the FSM is that studies have focused exclusively on negative outcomes, such as internalizing and externalizing problems [7, 15]. This myopic focus leads to a strong need for the examination of positive associations, such as positive developmental outcomes among children (e.g., social competence). The current study highlights the ways that family processes within the FSM promote desirable child outcomes, specifically focusing on the development of social competence.

Various studies have demonstrated the significant effects of family processes on social competence, primarily examining the individual relationships between different types of SES, marital conflict, parenting practices, and child mental health functioning, without considering associations in a comprehensive model. When considering the complex relationships among these variables, it is also important to consider independent associations, while controlling for other variables. For a more detailed exploration of the early protective factors potentially influencing diverse developmental maladjustment, the purpose of this preliminary study was to examine, in greater detail, social competence as a mediator of the relationships between SES, family processes, and children’s adjustment.

### Present study

Although several studies have demonstrated a significant impact of SES and family processes (i.e., marital conflict and parenting practices) on general adjustment among children, few have considered the relationship between child behavioral problems and SES, including family economic and parental educational levels, negative and positive aspects of marital conflict and parenting practices, and child social competence, in conjunction with one another. Most prior studies including the FSM have focused little attention on the educational domain of SES or the positive aspects of family functioning and child outcomes. When considering the complex relationships between these variables, it is important to consider independent associations, while controlling for other variables in a comprehensive model. Most studies have examined these complex relationships in a more piecemeal fashion, rarely integrating them into a unified conceptual model. Within the risk and resilience research framework, relational risk or protective factors are thought to make either additive or contingent contributions to adjustment.

**Fig. 1:**
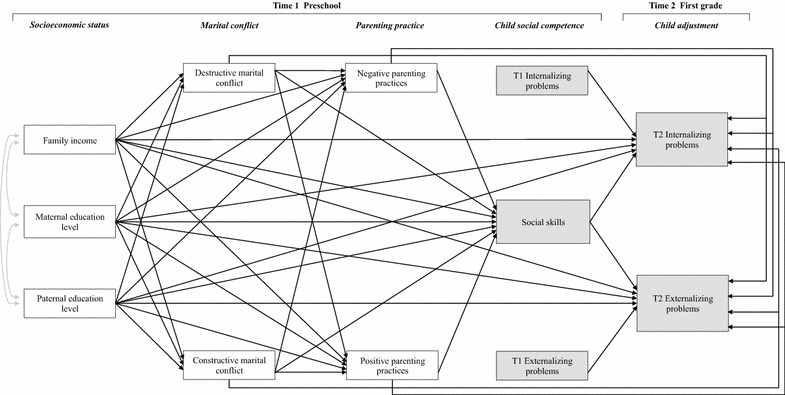
Hypothesized model. This model includes the hypothesized pathways among socioeconomic status, marital conflict, parenting practices, and children’s mental health functioning

Based on the observations above, the aim of this study was to clarify the roles of SES (i.e., family income and maternal and paternal educational levels), marital conflict (i.e., destructive and constructive marital conflict), parenting practices (i.e., negative and positive parenting practices), and child social competence (i.e., social skills) and behavioral problems (i.e. internalizing and externalizing problems), by analyzing these relationships in a comprehensive model. In the present study, we used longitudinal assessments of children’s externalizing and internalizing behaviors to evaluate the hypothesis that SES, marital conflict, and parenting practices predict children’s social competence, which is then related to later child adjustment. The mediational model in Fig. 1 was tested to estimate the direct effects of Time 1 (T1; participants were 5 years old, in preschool) SES, marital conflict, and parenting practices on Time 2 (T2; participants were 6 years old, in the first grade) behavioral problems, and to examine the indirect effects of T1 variables, through their effects on T1 social competence, on T2 behavioral problems. As a result, our study provides theoretical contributions to the FSM by incorporating additional critical factors (i.e., parental educational levels, positive aspects of family functioning, and positive child outcomes). Investigating the role of social competence as a mediating process in the link between relational risks such as SES and later child adjustment will enable important theoretical contributions to the understanding of processes involved in the development of adaptation among children with higher relational risks, and will provide implications for prevention and intervention efforts.

We hypothesized the following pathways: (1) SES indicators (i.e., family income and maternal and paternal educational levels) are, as predictors, differentially associated with family processes (i.e., marital conflict and parenting practices) and child mental functioning (i.e., social competence and adjustment) through distinct pathways; (2) both negative and positive aspects of family processes will mediate the relationship between SES and child mental health functioning; and (3) social competence in preschool, which is influenced by multidimensional family factors, will reduce the risk of behavioral problems in the first grade.

## Methods

### Participants

**Fig. 2:**
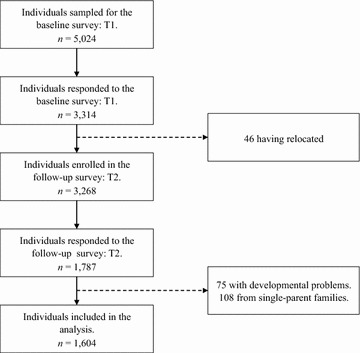
Flow chart of the study participants of the study

The current investigation consisted of two waves of data, taken 1 year apart, and was part of a longitudinal study that examined the influence of family factors on child social developmental outcomes. Figure 2 illustrates the flow chart of participants for this study. At T1 in 2014, participants were 5 years old and in preschool. Self-reported questionnaires were provided to the parents of children (*n* = 5024) enrolled in 52 kindergartens and 78 nursery schools in Nagoya city, which is a major urban area in Japan. A total of 3314 parents completed the questionnaires. At T2 in 2015, participants were 6 years old and in the first grade. Parents returned 1 year (12 months) after T1 to participate in the second wave of data collection. The retention rate from T1 to T2 was 53.9%, resulting in an ultimate sample size of 1787 for the current study.

In the present paper, to clarify the associations between SES accurately, including parents’ educational levels, marital relationship, parenting practices, and child developmental outcomes, the following individuals were excluded from analyses: (1) children from single-parent families, (2) children diagnosed with developmental problems, and (3) children whose mothers did not return completed questionnaires. For inclusion in this study, parents did not have to be the target child’s biological parent; however, they did need to reside with the child. For both T1 and T2, of the 1787 children, 1604 (89.8%) met the inclusion criteria. The children’s data, as provided by the mothers, were analyzed in this study.

At T1, mean age was 6.09 years (*SD* = .30), with 51.5% of the sample being males (*n* = 826) and 48.5% being females (*n* = 778). In total, 48.5% of the sample were children attending kindergarten (*n* = 778), and 51.5% were children attending nursery schools (*n* = 826). The mean ages of the mothers and fathers were 37.41 (*SD* = 4.47) and 39.33 (*SD* = 5.44) years, respectively. SES indicators (i.e., family income and parental education level) are shown in Table 1. The median household income was between Ұ 5,000,000 and Ұ 5,999,999 per year (approximately $ 50,000 and $ 59,999 USD per year). On average, mothers and fathers had completed comparable years of education, at 14.13 years (*SD* = 1.75) and 14.56 years (*SD* = 2.25), respectively.

**Table 1:** Parent and family characteristics of the study sample in percentages (*n* = 1604)

Description	n	%
Annual household income (in millions of yen)		
< 4	284	17.7
4–5	536	33.4
6–7	368	22.9
8–9	185	11.5
10–11	107	6.7
≥ 12	86	5.4
No response	38	2.4
Maternal education level		
Compulsory education (9 years)	35	2.2
Upper secondary school (12 years)	370	23.1
Less than 4 years at college/university (13–15 years)	661	41.2
Over 4 years at college/university (≥ 16 years)	529	33.0
No response	9	.6
Paternal education level		
Compulsory education (9 years)	77	4.8
Upper secondary school (12 years)	382	23.8
Less than 4 years at college/university (13–15 years)	239	14.9
Over 4 years at college/university (≥ 16 years)	895	55.8
No response	11	.7

We compared the T2 non-returning participants with the T2 returning participants on demographic features (i.e., parental age, family income, and parental education level). The mean ages of T2 non-returning participant mothers and fathers were 36.79 (*SD* = 4.82) and 38.92 (*SD* = 5.86) years, respectively. The T2 non-returning participants were comparatively younger parents that returned at T2, according to independent samples *t* tests (*p* < .05). A Chi square test yielded a significant (*p* < .001) difference between household incomes, with 24.8% of the T2 non-returning participants reporting below Ұ 3,999,999 per year, while only 17.7% of T2 returning participants reported this level. On average, the T2 non-returning participants’ mothers and fathers had comparable years of completed education, at 13.72 years (*SD* = 1.87) and 14.01 years (*SD* = 2.42), respectively. Additionally, a *t*-test revealed that the education level of non-returning participants was significantly lower (*p* < .001) than the education level of individuals that did return. Thus, the non-returning participants tended to have relatively lower SES than did returning participants, meaning that there was a lower response rate of individuals with low SES compared to high SES.

#### Ethics statement

The children’s parents and teachers were informed of the study’s purpose and procedures, and they were made aware that they were not obligated to participate. The teachers provided their written informed consent, and the parents submitted the same on behalf of their children prior to participating in this research. Ethical approval for this study was obtained from Kyoto University’s Ethics Committee in Kyoto, Japan (E2322).

### Measures

All the questions used for the self-developed questionnaire were questions translated into Japanese.

#### Predictors

##### Socioeconomic status

At T1, SES was defined as information about family income levels, as provided by the parents, and parental education. Parents were asked to report their total yearly family income, their education in years, and their completed education levels by choosing one of the following response options: compulsory education (9 years), vocational upper-secondary school/general upper-secondary school (12 years), less than 4 years at college/university (13–15 years; i.e., junior college, vocational school, or professional school), and over 4 years at college/university (≥ 16 years). Each of the SES scores (i.e., yearly family income and years of parental education) were converted to *z* scores.

#### Mediators

##### Marital conflict

At T1, the Quality of Co-parental Communication Scale (QCCS), a 10-item self-report questionnaire, was used to assess each parent’s feelings or behaviors within the context of the co-parenting relationship [120]. This measure is composed of the following two subscales: Co-parental Conflict (four items relating to conflict, hostility, tension, and disagreements) and Co-parental Support (six items relating to accommodation, helpfulness, and resourcefulness). Items are rated on a 5-point Likert scale ranging from 1 (*Never*) to 5 (*Always*). The Conflict and Support subscales assess parents’ perceptions of the co-parenting relationship. The Conflict subscale measures the negative aspect of the co-parenting relationship, with higher conflict scores indicating more co-parental communication conflict [152]. In the current study, we considered Co-parental Conflict as destructive conflict. Conversely, the Support subscale measures positive aspects of the co-parenting relationship, with higher support scores indicating more supportive co-parental communication [152]. Specifically, the Support subscale measures “general support” including helpfulness, resourcefulness, and cooperation [152], as opposed to the constructive aspects of conflict. However, in the current study, we considered Co-parental Support as constructive marital conflict. The scales have adequate internal consistency and construct validity [152–154]. The internal consistency was .88 and .74 for Conflict and Support scales, respectively [152]. The current study found internal consistencies of .77 and .86 for the Conflict and Support scales, respectively. Each QCCS total score was converted to a *z* score.

##### Parenting practice

At T1, the Alabama Parenting Questionnaire (APQ), a 42-item self-report questionnaire, was used to assess various aspects of parenting behavior [155, 156]. The measure is composed of the following five subscales: Poor Monitoring/Supervision, Inconsistent Discipline, Corporal Punishment, Positive Parenting, and Involvement. Items are rated on a 5-point Likert scale ranging from 1 (*Never*) to 5 (*Always*). Participants self-reported their own parenting behavior. The developers have reported that the measure has adequate internal consistency and construct validity [156]. The internal consistency of the subscales ranges from .46 to .80 [156]. In this study, the subscales’ internal consistency ranged from .71 to .76.

In this study, we standardized the separate positive and negative parenting composite scores [157]. Scores on the Poor Monitoring/Supervision, Inconsistent Discipline, and Corporal Punishment subscales of the APQ were combined to form a negative parenting composite score, whereas scores on the Positive Parenting and Involvement subscales were combined to form a Positive Parenting composite score. The Negative Parenting composite score was calculated by converting the Poor Monitoring/Supervision, Inconsistent Discipline, and Corporal Punishment subscale scores to *z* scores and then averaging them, with higher scores indicating more negative parenting. Similarly, the Positive Parenting composite score was calculated using the same method for the Positive Parenting and Involvement subscale scores, with higher scores indicating more positive parenting.

##### Child social competence

At T1, the Social Skills Questionnaire (SSQ) was used as an index of observer ratings of child social competence. In the current study, the children’s teachers evaluated their social skills using this scale. The SSQ is a 24-item measure of children’s social competence in relation to “cooperation”, “self-control”, and “assertion” [158–160], as factors affecting social adaptation in later life [90]. These clusters of social behaviors can briefly be characterized as follows: Cooperation—behaviors such as helping others, sharing with a peer, and complying with rules such as sharing and obeying; Self-control—behaviors that emerge in conflict situations, such as responding appropriately to (i.e., controlling one’s temper) teasing or corrective feedback from an adult; and Assertion—behaviors such as asking others for help/information and responding to others’ actions (e.g., responses to peer pressure).

The SSQ has the following three subscales: Cooperation (eight items; e.g., the child helps someone voluntarily), Self-control (eight items; e.g., the child behaves if there is a need), and Assertion (eight items; e.g., the child initiates a conversation with someone). These factors are based upon, and positively correlated with, the Social Skills Rating System (SSRS) [90], which is one of the most widely used social skills scales and was used in the National Institute of Child Health and Human Development (NICHD) study [161, 162]. The SSQ’s items are rated on a 3-point scale ranging from 0 (*Not at all*) to 2 (*Often*), yielding total scores for cooperation, self-control, and assertiveness. The SSQ has adequate internal consistency and construct validity; the subscales’ internal consistency has previously ranged from .91 to .93 [158], with a range from .84 to 94 in the current study. Furthermore, the present study combined total scores for cooperation, self-control, and assertiveness to form a social skills score, with higher scores indicating better social skills. The social skills score was calculated by converting scores on the Cooperation, Self-control, and Assertion subscales to *z* scores, and then averaging them.

#### Criterion variables

##### Child adjustment

The Strengths and Difficulties Questionnaire (SDQ) is a 25-item measure of parents’ perceptions of their children’s prosocial and difficult behaviors, and it is designed to assess general internalizing and externalizing emotional and behavioral problems [163]. In this study, children’s mothers evaluated their behavioral adjustment using this scale at both T1 and T2. The measure is composed of the following five subscales: Emotional Symptoms, Conduct Problems, Hyperactivity-Inattention, Peer Problems, and Prosocial Behavior. Items were rated on a 3-point Likert scale ranging from 0 (*Not true*) to 2 (*Certainly true*). The scales’ internal consistency and construct validity were reported as adequate [164–166].

In this study, the Emotional Symptoms and Peer Problems subscales of the SDQ were combined to form an Internalizing Problems scale (Cronbach’s α = .65, .71), while the Conduct Problems and Hyperactivity-Inattention subscales were combined to form an Externalizing Problem scale (Cronbach’s α = .74, .77), as suggested by Goodman et al. [167], with higher scores indicating more behavioral problems. Each SDQ total score was converted to a *z* score.

### Procedure

To conduct our study, we asked the kindergartens and nursery schools with 50 or more students, in Nagoya city, to participate. As a result, principals of 130 facilities (52 kindergartens and 78 nursery schools) gave us permission to conduct our survey and meet with participating parents. To recruit families at T1, self-reported questionnaires were distributed at the participating facilities to all parents of 5 year olds (*n* = 5024). Participants received an information sheet and questionnaires on childrearing, in relation to family factors (i.e., SES, family relationships, and parenting style), and child behavioral adjustment (i.e., externalizing and internalizing problems). Participants provided written informed consent and agreed to participate. The parents completed the questionnaires at a single time point and returned these to participating facilities in sealed envelopes to prevent teachers from seeing the questionnaires. Then, the teachers evaluated the children’s social skills using the SSQ. All sealed envelopes containing questionnaires and SSQ evaluations were returned to the researcher from the respective principals.

At T2, 12 months later, participants were contacted again when the children were in the first grade. At T1, the researcher obtained the address of participants, and, at T2, the researcher mailed the participants questionnaires on childrearing in relation to family factors and child behavioral adjustment. Participants who completed the questionnaires returned them to the researcher by mail. Access to the data was restricted to the researchers of the current longitudinal study.

### Data analyses

First, prior to developing a model of the relationships among SES, parental relationship, parenting practices, and child social competence and adjustment, correlation analyses were utilized to determine the associations among SES (i.e., T1 family income, maternal and paternal levels of education), marital relationship (i.e., T1 destructive and constructive marital conflict), parenting practices (i.e., T1 negative and positive parenting practices), child social competence (i.e., T1 social skills), and child adjustment (i.e., T1 and T2 internalizing and externalizing problems).

Second, path analyses were conducted to estimate direct and indirect paths between SES, parental relationship, parenting practices, and child social competence and adjustment. Structural equation modeling analyses were conducted using full information maximum-likelihood estimation in the presence of missing data. The hypothesized model is presented in Fig. 1. In the models, SES (i.e., T1 family income and parental level of education) was specified as a predictor of the marital relationship (i.e., T1 destructive and constructive marital conflict), parenting practices (i.e., T1 negative and positive parenting practices), child social competence (i.e., T1 social skills), and behavioral adjustment (i.e., T1 and T2 externalizing and internalizing problems). We estimated how family factors (i.e., SES, marital conflict, and parenting) and child social competence in preschool influenced the children’s behavioral adjustment in the first grade. The model also included T1 behavioral adjustment as control variables; through controlling for initial levels of maladjustment, the model would appropriately address changes in behavioral adjustment. Based on previous findings in the literature, we expected the effect of T1 SES indicators on T2 behavioral adjustment to be mediated by the T1 parental relationship, parenting practices, and social competence. Moreover, we expected an inverse effect between T1 social competence and T2 adjustment.

To assess fit, we examined the Comparative Fit Index (CFI) [168], the Incremental Fit Index (IFI) [169], and the Root Mean Square Error of Approximation (RMSEA) [170]. Good model fit is reflected in CFI and IFI values above .90 [168, 169]. Regarding the RMSEA, good fit was represented by a value smaller than .05 and reasonable fit was represented by values ranging from .05 to .08 [171]. All the statistical analyses were conducted using SPSS version 23.0 and Amos version 23.0.

## Results

### Preliminary analyses

SES indicators are shown in Table 1. Other descriptive statistics for all variables measured by the scales (i.e., marital conflict, parenting practices, child social competence, and behavioral adjustment) are presented in Table 2. A correlation matrix of the SES indicators, marital conflict, parenting practices, and child social competence and behavioral adjustment is shown in Table 3. Analyses in study composites showed that all correlations of the study composites were statistically significant. The indicators of SES, marital conflict, parenting practice, and child social competence and behavioral adjustment were interrelated, supporting our hypotheses and previous empirical findings. Each SES variable (i.e., family income and maternal and paternal educational levels) was negatively related to destructive marital conflict, negative parenting, and the children’s externalizing and internalizing behavioral problems. Conversely, it was positively related to constructive marital conflict, positive parenting, and children’s social skills. In turn, social skills inversely correlated with children’s externalizing and internalizing behavioral problems.

**Table 2:** Descriptive statistics for the study variables (*n* = 1604)

Description	Range	M	SD	Cronbach’s α
Marital conflict: Quality of Co-Parental Communication Scale (QCCS)				
Co-parental Conflict	4–20	9.88	3.01	.77
Co-parental Support	6–30	25.16	4.14	.86
Parenting practice: Alabama Parenting Questionnaire (APQ)				
Poor monitoring/supervision	10–50	12.87	2.94	.71
Inconsistent discipline	6–30	14.53	3.77	.73
Corporal punishment	3–15	7.06	2.17	.72
Positive parenting	6–30	22.35	3.49	.76
Involvement	10–50	37.99	5.07	.75
Social competence: Social Skills Questionnaire (SSQ)				
Cooperation	0–16	10.97	4.13	.94
Self-control	0–16	14.18	2.64	.90
Assertion	0–16	14.08	2.37	.84
Child adjustment: Strengths and Difficulties Questionnaire (SDQ)				
T1 internalizing problems	0–20	3.34	2.70	.65
T1 externalizing problems	0–20	5.02	3.21	.74
T2 internalizing problems	0–20	3.88	3.04	.71
T2 externalizing problems	0–20	5.15	3.29	.77

T1: Time 1, preschool; T2: Time 2, first grade

**Table 3:** Correlations among socioeconomic status, marital conflict, parenting practice, and child social competence and adjustment (*n* = 1604)

Variable	1	2	3	4	5	6	7	8	9	10	11	12
Time 1—Preschool												
Socioeconomic status												
1. Family income	–											
2. Maternal education level	.33***	–										
3. Paternal education level	.30***	.42***	–									
Marital conflict												
4. Destructive marital conflict	− .14***	− .10***	− .14***	–								
5. Constructive marital conflict	.13***	.13***	.14***	− .62***	–							
Parenting practice												
6. Negative parenting practices	− .17***	− .14***	− .14***	.24***	− .16***	–						
7. Positive parenting practices	.14***	.13***	.09***	− .18***	.28***	− .21***	–					
Child social competence												
8. Social skills	.18***	.15***	.16***	− .24***	.23***	− .20***	.17***	–				
Child adjustment												
9. T1 internalizing problems	− .14***	− .12***	− .09***	.23***	− .19***	.19***	− .13***	− .35***	–			
10. T1 externalizing problems	− .16***	− .12***	− .14***	.22***	− .19***	.36***	− .25***	− .44***	.36***	–		
Time 2—First grade												
Child adjustment												
11. T2 internalizing problems	− .18***	− .19***	− .08***	.23***	− .21***	.19***	− .17***	− .44***	.64***	.33***	–	
12. T2 externalizing problems	− .19***	− .18***	− .17***	.24***	− .24***	.31***	− .24***	− .53***	.32***	.74***	.48***	–

[Marital relationship] Destructive marital conflict: QCCS Co-parental Conflict; constructive marital conflict: QCCS Co-parental Support. [Parenting practice] Negative parenting practices: APQ Poor monitoring/supervision, Inconsistent discipline, Corporal punishment; positive parenting practices: APQ Involvement, Positive parenting. [Social competence] Social skills: SSQ Cooperation, Self-control, Assertion

* *p* < .05; ** *p* < .01; *** *p* < .001

### Mediational models for SES, marital conflict, parenting practices, child social skills, and child adjustment

**Fig. 3:**
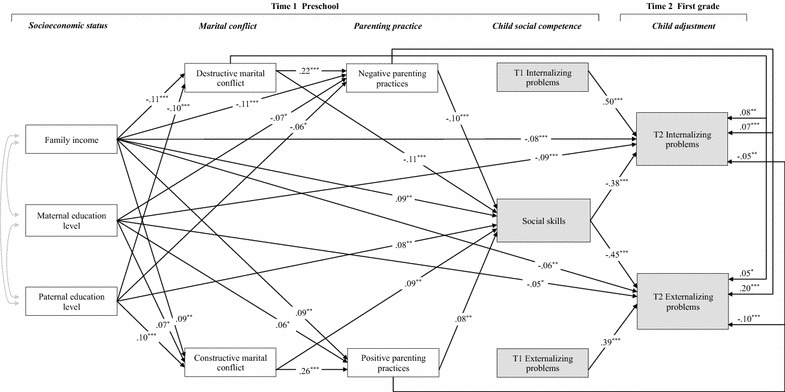
Statistically significant paths. This model includes the paths that were statistically significant in the hypothesized model. Model fit statistics: χ^2^ (18) = 31.89; CFI = .99; IFI = .99; RMSEA = .02. *p < .05; **p < . 01; ***p < .001

Longitudinal models examined the impact of SES, marital conflict, and parenting practices on child social competence and behavioral adjustment (Hypothesized model; Fig. 1). Figure 3 depicts the final path models, and the path diagram specifies both direct and indirect paths linking T1 SES indicators (i.e., family income and maternal and paternal educational levels) to T2 child behavioral adjustment (i.e., externalizing and internalizing problems; Table 4).

**Table 4:** Path analyses (*n* = 1604)

Construct			B	SE	β
Socioeconomic status					
Family income	→	Destructive marital conflict	− .11	.03	− 3.83***
Family income	→	Constructive marital conflict	.09	.03	3.04**
Family income	→	Negative parenting practices	− .11	.03	− 3.82***
Family income	→	Positive parenting practices	.09	.03	3.05**
Family income	→	Social skills	.09	.03	3.29**
Family income	→	T2 internalizing problems	− .08	.02	− 3.48***
Family income	→	T2 externalizing problems	− .06	.02	− 2.65**
Maternal education level	→	Destructive marital conflict	− .03	.03	− .90
Maternal education level	→	Constructive marital conflict	.07	.03	2.17*
Maternal education level	→	Negative parenting practices	− .07	.03	− 2.28*
Maternal education level	→	Positive parenting practices	.06	.03	2.03*
Maternal education level	→	Social skills	.04	.03	1.18
Maternal education level	→	T2 internalizing problems	− .09	.02	− 3.75***
Maternal education level	→	T2 externalizing problems	− .05	.02	− 2.11*
Paternal education level	→	Destructive marital conflict	− .10	.03	− 3.46***
Paternal education level	→	Constructive marital conflict	.10	.03	3.31***
Paternal education level	→	Negative parenting practices	− .06	.03	− 2.08*
Paternal education level	→	Positive parenting practices	.01	.03	.38
Paternal education level	→	Social skills	.08	.03	2.85**
Paternal education level	→	T2 internalizing problems	−.04	.02	−1.69
Paternal education level	→	T2 externalizing problems	− .02	.02	− .67
Marital conflict					
Destructive marital conflict	→	Negative parenting practices	.22	.03	6.83***
Destructive marital conflict	→	Positive parenting practices	−.01	.03	−.30
Destructive marital conflict	→	Social skills	− .11	.03	− 3.47***
Destructive marital conflict	→	T2 internalizing problems	.08	.03	3.08**
Destructive marital conflict	→	T2 externalizing problems	.05	.02	2.25*
Constructive marital conflict	→	Negative parenting practices	−.01	.03	−.19
Constructive marital conflict	→	Positive parenting practices	.26	.03	8.16***
Constructive marital conflict	→	Social skills	.09	.03	2.68**
Constructive marital conflict	→	T2 internalizing problems	− .03	.03	− 1.09
Constructive marital conflict	→	T2 externalizing problems	− .01	.02	− .54
Parenting practice					
Negative parenting practices	→	Social skills	− .10	.03	− 3.90***
Negative parenting practices	→	T2 internalizing problems	.07	.02	3.30***
Negative parenting practices	→	T2 externalizing problems	.20	.02	10.08***
Positive parenting practices	→	Social skills	.08	.03	3.10**
Positive parenting practices	→	T2 internalizing problems	− .05	.02	− 2.56**
Positive parenting practices	→	T2 externalizing problems	− .10	.02	− 4.90***
Child social competence					
Social skills	→	T2 internalizing problems	− .38	.02	− 18.65***
Social skills	→	T2 externalizing problems	− .45	.02	− 22.75***
Child adjustment					
T1 internalizing problems	→	T2 internalizing problems	.50	.02	27.03***
T1 externalizing problems	→	T2 externalizing problems	.39	.02	21.30***

* *p* < .05; ** *p* < .01; *** *p* < .001

The standardized coefficients are shown in Fig. 3. Model fit was tested with multiple indices; the model provided a good fit to the data [χ^2^ (18) = 31.89, *p* = .023; CFI = .99; IFI = .99; RMSEA = .02].

In the model, several statistically significant direct and indirect paths were found between the predictors and criterion variables. Family income was found to be a significant predictor of lower levels of destructive marital conflict (β = − .11, *p* < .001), lower levels of negative parenting practices (β = − .11, *p* < .001), higher levels of constructive marital conflict (β = .09, *p* < .01), higher levels of positive parenting practices (β = .09, *p* < .01), higher levels of child social skills (β = .09, *p* < .01), and lower levels of T2 internalizing problems (β = − .08, *p* < .001) and T2 externalizing problems (β = − .06, *p* < .01). The indirect paths from family economy to child mental health functioning (i.e., social skills and internalizing and externalizing problems) through marital conflict and parenting practices were also significant.

Maternal education level was found to be a significant predictor of lower levels of negative parenting practices (β = − .07, *p* < .05), higher levels of constructive marital conflict (β = .07, *p* < .05), higher levels of positive parenting practices (β = .06, *p* < .05), and lower levels of T2 internalizing problems (β = − .09, *p* < .001) and T2 externalizing problems (β = − .05, *p* < .05). The indirect paths from maternal education level to child mental health functioning (i.e., social skills and internalizing and externalizing problems) through marital conflict and parenting practices were also significant.

Paternal education level was found to be a significant predictor of lower levels of destructive marital conflict (β = − .10, *p* < .001), lower levels of negative parenting practices (β = − .06, *p* < .05), higher levels of constructive marital conflict (β = .10, *p* < .001), and higher levels of child social skills (β = .08, *p* < .01). The indirect paths from paternal education level to child mental health functioning (i.e., social skills and internalizing and externalizing problems) through marital conflict and parenting practices were also significant.

Notably, in terms of the negative dimension of family processes (marital conflicts and parenting practices), T1 destructive conflict was directly, negatively related to social skills (β = − .11, *p* < .001), and indirectly, negatively related to T1 social skills through T1 negative parenting practices. T1 negative parenting practices were directly, negatively related to social skills (β = − .10, *p* < .001). Regarding the positive dimension of family processes, T1 constructive conflict was directly, positively related to social skills (β = .09, *p* < .01), and indirectly, positively related to T1 social skills through T1 positive parenting practices. T1 positive parenting practices were directly, positively related to social skills (β = .08, *p* < .01). In turn, T1 social skills were found to be a direct and significant predictor of lower levels of T2 internalizing problems (β = − .38, *p* < .001) and T2 externalizing problems (β = − .45, *p* < .001), while controlling for behavior problems at T1.

Therefore, consistent with the hypotheses, each SES indicator was significantly and independently associated with child mental health functioning (i.e., social skills and internalizing/externalizing problems) through positive and negative dimensions of marital conflict and parenting practices. Notably, T1 social skills in preschool, which were affected by T1 family factors, predicted lower levels of T2 behavioral problems in the first grade.

## Discussion

Our longitudinal study explored, in a comprehensive model, marital conflict (i.e., constructive and destructive marital conflict), parenting practices (i.e., positive and negative parenting practices), and social competence (i.e., social skills) as mediators of the association between SES (i.e., family income, maternal and paternal educational levels) in preschool and child behavioral adjustment (i.e., internalizing and externalizing problems) in the first grade. Our extension of previous research investigating the relationships between SES and child behavioral adjustment comprised the following three points. (1) We included both family income, and maternal and paternal education levels as SES indicators, and as predictors of family processes (i.e., marital conflict and parenting practice) and mental health functioning of children (i.e., social competence and behavioral adjustment), in a unified model. We expected each SES indicator, as predictors, to be differentially associated with family processes and child mental functioning through distinct pathways. (2) We included not only negative mediators (i.e., destructive marital conflict and negative parenting practices), but also positive mediators (i.e., constructive marital conflict and positive parenting), as mediating mechanisms in the link between SES and child mental health functioning. We expected both negative pathways (negative spillover) and positive pathways (positive spillover) in the family process model. (3) We included not only negative child developmental outcomes (i.e., behavioral problems), but also desirable child developmental outcomes (i.e., social competence) in the relationship between family factors (i.e., SES and family processes) and child mental health functioning. Moreover, we focused on social competence as a mediator of the relationship between family factors and child behavioral problems. We expected social competence in preschool, which was affected by different types of family factors, to be inversely related to the symptoms of behavioral problems in the first grade.

Our main findings were the following. (1) Family income and parental education levels were differentially associated with child mental health functioning through distinct pathways. This result provides evidence that lower SES (i.e., lower family income and lower parental education level) is both directly and indirectly associated with more destructive marital conflict, more use of negative parenting practices, less constructive marital conflict, less use of positive parenting practices, poorer social competence, and more symptoms of behavioral problems. This suggests that, by contrast, higher SES (higher family economy and higher parental education levels) is both directly and indirectly associated with less destructive marital conflict, less use of negative parenting practices, more constructive marital conflict, more use of positive parenting practices, higher social competence, and fewer symptoms of behavioral problems. (2) We identified both negative and positive pathways between SES and child mental health functioning. Positive mediators included constructive marital conflict and positive parenting practices. This result suggests that destructive marital conflict is indirectly and negatively related to child mental health functioning through negative parenting practices in the relationship between SES and child mental health functioning. Simultaneously, in that relationship, destructive marital conflict was directly and negatively related to child mental health functioning. By contrast, these results indicate that constructive marital conflict demonstrates an indirect and positive relationship to child mental health functioning through positive parenting practices, as well as a direct positive relationship to child mental health functioning. (3) Social skills, which were associated with different types of family factors (i.e., SES, including family income and parental education levels, and both negative and positive dimensions of family processes), adversely affected later internalizing and externalizing behaviors. This result suggests social skills were lowered by the negative aspects of family processes (i.e., destructive marital conflict and negative parenting practices) and raised by the positive aspects of family processes (constructive marital conflict and positive parenting practices) in preschool, which reduced later symptoms of internalizing and externalizing problem behaviors in the first grade. That is, social skills in preschool played a potentially protective role in preventing later behavioral problems. Therefore, our longitudinal analysis supported the initial hypotheses.

### Path of family economic situation, family processes, and child mental health functioning

In this study, family income was directly linked to marital conflict, parenting practices, and in turn, child mental health functioning (i.e., social competence and behavioral problems). This result is consistent with previous research findings identifying a direct path of family income to destructive marital conflict and negative parenting practices, and in turn, child outcomes [7, 28–30, 35, 36, 63]. Furthermore, this result supports the FSM’s prediction that family income affects children’s socio-emotional development through its influence on parents’ psychological well-being and, therefore, the inter-parental relationship and parent/child interactions [15]. The result also supports the notion of negative spillover effects and is consistent with family systems theory [17, 18].

Conversely, we found a positive pathway within which a higher family economic status was associated with more constructive marital conflict, and in turn, more use of positive parenting practices, resulting in higher mental health functioning. This result supports the notion of the positive spillover effect, with the positive inter-parental relationship spilling over into the parent/child relationship, resulting in more positive parenting practices. Similar to negative spillover effects and consistent with family systems theory [18], positive emotions from inter-parental relationships may transfer to parent/child relationships [82, 83]. This result, that there is a positive spillover effect in the family process model, is an extension of previous studies.

Additionally, we found that family income was directly related to child mental health functioning (i.e., social competence and behavioral problems), while controlling for other variables. There are likely to be other factors that were not accounted for in our model. For example, the Family Investment Model (FIM), which is concerned with the advantages reaped by the developing child because of family wealth [28, 172, 173], may explain this association. The FIM proposes that families with more economic resources can make significant investments in the development of their children, whereas those with lower incomes must invest in more immediate family needs [1, 7, 174]. Income enables families to invest in building their children’s human capital. These investments in children involve several dimensions of goods and services, including parents’ direct and indirect stimulation of learning (e.g., providing learning materials and activities, and support through advanced training and schools), the family’s standard of living (e.g., adequate food, housing, clothing, medical care), and living in a more advantaged neighborhood environment that fosters a child’s development [7, 175, 176]. According to this perspective, children in disadvantaged families tend to fare worse because they have limited access to resources that help them develop. Mayer demonstrated that children in disadvantaged families lived under worse conditions, owned fewer stimulating materials, and were less likely to engage in stimulating activities [176]. After controlling for other family background characteristics, these resources were associated with children’s developmental outcomes [176]. Therefore, the apparent direct effect of family economic status found in the current study could possibly be mediated by factors that were not accounted for in our model. Future studies should investigate this possibility by including more family factors related to child mental health functioning in their models.

### Path of parental educational level, family processes, and child mental health functioning

As mentioned earlier, despite the many studies completed in this area, few studies have simultaneously investigated the influence of family income and maternal and paternal education levels as predictors in the relationships between SES, family processes, and child mental health functioning [6, 7, 28]. Although most of the previous FSM studies have focused primarily on economic conditions, we suspect that they tend to capture a limited scope of the influence of educational achievement. In this study, both maternal and paternal educational levels were independently linked with parental functioning and parent/child interactions, and in turn, with child mental health functioning in a unified model, while controlling for economic conditions. In addition, this result also supports the notion of both positive and negative spillover effects [17, 18], as educational levels were positively related to higher levels of constructive marital conflict, and in turn, higher levels of positive parenting, resulting in better developmental outcomes. Therefore, the results regarding the effects of multiple components of SES, including family income and maternal and paternal education levels on child mental health functioning through distinct pathways, are an extension of those found in previous studies.

In terms of the relationship between educational level and marital conflict, the results of the current study are consistent with those of previous research showing educational attainment to be inversely related to destructive marital conflict [62], and parental educational attainment to be positively related to greater marital satisfaction and marital stability [30, 63–65]. More precisely, paternal education was linked to both destructive and constructive conflict; however, maternal education was linked to only constructive conflict. This might be due to difference of effect of maternal and paternal education on decision-making in the home. As mentioned earlier, previous studies have suggested that higher education helps parents strengthen their communication and problem-solving skills, and promotes effective problem solving between parents [50, 58]. In addition, higher education tends to make fathers positively participate in decision-making in the home, whereas, fathers with lower education negatively participate [177–179]. Therefore, in this study, paternal education might more strongly affect both destructive and constructive than maternal education.

Furthermore, in terms of the relationship between educational level and parental involvement, we found that maternal education was associated with positive parenting practices, but not paternal education; however, both maternal and paternal education were linked to negative parenting practices. This result might indicate that the effects of parental education on involvement is larger for maternal education than for paternal. This might be due to mothers tending to be the main provider of care within the households of Japan. Many studies suggest that mothers assume the primary parenting role, in that mothers were found to be more intrusive toward father/child interactions [180–182]. In addition, this result is consistent with previous research findings. A large number of studies suggest higher maternal educational attainment to be positively related to positive parenting attitudes, such as talking to children warmly or supportively [72, 73], whereas lower educational levels have been found to be predictors of negative parenting, such as harshness and physical disciplinary tactics [33, 34, 183–185]. However, although many studies suggest maternal educational attainment is related to parenting attitudes, few studies have comparatively investigated the influence of paternal education levels on parental involvement. These results imply the possibility that both maternal and paternal educational levels are independently related to parenting attitudes.

One of the important mechanisms in the effect of parental education levels on family processes and children’s development is likely to be parental knowledge about childrearing and child development. Lower levels of parental education are associated with negative parenting attitudes, such as physical and authoritarian disciplinary tactics [33, 34, 183–185]. It has been suggested that this is due to a lack of knowledge concerning the counterproductive outcomes of severe disciplinary responses and appropriate alternatives to harsh discipline [33, 183]. Higher levels of parental education have also been positively associated with sensitivity, positive regard, and cognitive stimulation of children [186]. Further, it has been suggested that higher educational levels are associated with increased knowledge about childrearing and child development, and more supportive parenting [72, 73]. Therefore, both maternal and paternal education levels may influence parenting attitudes, even when controlling for family income, whereas educational attainment affects parenting attitudes through the adverse effects of poor family economic situations on parents’ mental well-being. Therefore, we assume that findings related to economic predictions based on the FSM are likely to reflect educational differences in SES as well. Educational levels are likely to play an important role in the relationships among SES, family processes, and child mental health functioning.

In addition, we found a direct association between parental education levels and child mental health functioning (i.e., social competence and behavioral problems), while controlling for other variables. There are likely to be other factors that were unaccounted for in our model. The FIM may also explain this mediating pathway to provide evidence for the plausibility of parental education level as an important aspect of the investment process [1, 7].

The model proposes that, similar to family income, parental education level has an influence on parental investments, and that these investments, in turn, will have a positive relationship with child development. Parents with higher education levels acquire more knowledge about child development, have a greater understanding of strategies to encourage social competence, and may be more effective in teaching children [72, 73, 187]. Families with higher educational levels and more knowledge about childrearing and child development may be more willing to make significant investments in their children’s development. Despite the reasonableness of this hypothesized mediating process, there have been limited investigations into the impact of parental education level, in terms of the FIM.

However, some evidence is consistent with the aforementioned ideas. For example, a previous study found education level to be positively correlated with parental investments involving a more enriched and positive child-rearing environment, characterized by the availability of play and learning materials, and the organization and diversity of the physical environment [188].

Investment in this regard is not only material (e.g., reading materials, learning materials, neighborhood, health insurance, and quality of residence), but also emotional (e.g., parenting beliefs and behaviors) [189]. For example, more highly educated parents create a richer and more complex language environment for their children [190]. They also spend more time communicating with their children [173, 191]. A previous study found parental education to be positively related to children’s language skills, including vocabulary and reading skills [192]. The richness of the language environment in inter-parental and parent/child interactions may mediate the association between parents’ education levels and a child’s productive vocabularies, and enhance the children’s social competence. Therefore, there are likely to be other factors in family processes that were unaccounted for in our model. This result is likely to support the FIM, including its suggestion of parental educational attainment as an SES indicator.

More precisely, regarding the path between parental education and social competence, we found that paternal education was directly linked to social competence, but maternal education was not. There are likely to be other factors of paternal characteristic roles that were unaccounted for in our model, in addition to factors of the FIM. For instance, paternal involvement tends to be more physical and challenging than maternal [193, 194]. Physical and challenging play is an important component of human socialization [195, 196]. Father/child physical play is likely to help children learn to regulate their own behavior, and practice coping with failure or frustration and interpreting others’ emotions. This is because father/child physical play has been linked to children’s emotion-regulation and peer competence [196–199]. The positive association between father/child physical play and child social competence is a common empirical finding [195, 200–203]. In addition, several studies have suggested that fathers with higher educational levels tend to be more involved, have more positive engagement, and are more accessible to their children [78–80]. Therefore, fathers with higher educational levels might promote child social competence through not only factors of FIM, but also characteristic parental involvement, such as physical and challenging play.

Moreover, regarding the path between parental education and behavioral problems, we found that maternal education was both directly and indirectly linked to T2 internalizing behavior and externalizing behavior; however, the link for paternal education was only indirect. There are also likely to be other factors of maternal characteristic roles that were unaccounted for in our model. For instance, mothers with higher education tend to have higher quality of mother/child interactions, such as sensitivity and responsiveness [188, 204]. Past researchers have found that maternal sensitivity and responsiveness significantly shape children’s cognitive development. Furthermore, cognitive competence deficits have also been reported as a vulnerability factor in causing behavioral problems [205–208]. Therefore, maternal educational achievement might affect behavioral problems through the effect of specific mother/child interactions.

Future studies should investigate the possibilities of the direct effect of parental education levels, as found in this study, being mediated by factors not accounted for in our model. This could be done by including more factors in future models.

### The role of social competence in the relationships among SES, family processes, and adjustment

We focused on both negative child developmental outcomes (i.e., behavioral problems) and desirable child developmental outcomes (i.e., social competence) in the relationship between family factors (i.e., SES, marital conflict, and parenting practices) and child mental health functioning. We also highlighted the ways that family processes within the FSM promote positive developmental outcomes.

In the current study, social competence mediated the association between family factors and children’s behavioral adjustment in a comprehensive model. SES was positively related to social competence and inversely related to internalizing and externalizing symptomatology, through positive and negative dimensions of parents’ marital relationships and parenting styles. This result is an extension of those of previous studies, in which multidimensional family factors (i.e., SES, marital conflict, and parenting style) were related to both negative and positive outcomes in a comprehensive model. This result is consistent with several previous research findings identifying the direct individual path within which marital conflict and parenting practices are associated with child mental health functioning.

In terms of parenting practices and child mental health functioning, in this study, negative parenting practice was directly linked with poorer mental health functioning (i.e., poorer social skills, and more internalizing and externalizing problems). By contrast, positive parenting was directly linked to higher mental health functioning (i.e., better social skills and fewer internalizing and externalizing problems). Previous studies have suggested that negative parenting behaviors, such as harsh discipline, being emotionally neglectful, or demonstrating rejecting behaviors, are often associated with lower sociability-competence and increased problem behaviors in children [16, 25, 143], while positive parenting behaviors, such as emotional expressiveness, responsiveness, and support, have been shown to predict better empathy and social functioning in children [140–143].

Additionally, in terms of marital conflict and child mental health functioning, in this study, marital conflict was not only indirectly related to child outcomes through parenting practices, but also directly related to child outcomes. Parents’ destructive marital conflict was directly linked with poorer mental health functioning (i.e., poorer social skills, and more symptoms of internalizing and externalizing problems). By contrast, parents’ constructive conflict was directly linked to better mental health functioning (i.e., better social skills), and in turn, fewer symptoms of behavioral problems. These results are consistent with previous studies indicating that exposure to marital conflict is associated with different responses in children, depending on the type of inter-parental relationship [146, 209].

Many previous studies have shown that destructive marital conflict negatively affects social competence [144]. In addition, the relationships between inter-parental destructive conflict and negative psychological adjustment among children (e.g., internalizing symptoms and externalizing problems) are well established [146, 149, 209–211]. That is, destructive marital conflict has been shown to adversely influence children’s social competence [212–215], internalizing symptoms [211, 216], and externalizing problems [210, 211]. However, limited research has investigated the impact of constructive marital conflict on child mental health functioning. Therefore, the current result is an extension of those in previous studies, which demonstrated constructive marital conflict’s direct association with child social development.

One of the important direct mechanisms of the effect of inter-parental relationship on children’s development is likely to be modeling. According to social learning theory, children’s social development can be influenced by modeling the behaviors and attitudes of significant persons in their lives, such as parents [217]. Child social development may be both positively and negatively related to parents’ social development, due to the effects of modeling [218–220]. Consistent with the modeling mechanism proposed by the spillover hypothesis, children may directly model conflict behavior exhibited by their parents. In the case of destructive marital conflict, children whose parents resolve their problems through aggressive behavior are more likely to learn that aggression is an acceptable way of dealing with disagreements, and thus, may act aggressively when interacting with their peers [149, 221, 222]. Therefore, destructive marital conflict is likely to directly limit children’s social development. By contrast, in the case of constructive marital conflict, children whose parents resolve problems through supportive cooperation are more likely to learn from the negotiations between their mothers and fathers during the decision-making process, allowing them a blueprint to communicate more effectively and efficiently when interacting with their peers [150]. Therefore, constructive marital conflict is likely to directly enhance social development.

In addition, in this study, social skills in preschool, which were affected by family factors, inversely predicted later internalizing and externalizing symptomatology in the first grade, after controlling for preschool behavioral symptomatology. This result is consistent with previous research. A number of studies have shown negative correlations between social competence and behavioral problems. Early social competence among children is an important predictor of later social adjustment and psychopathology [223–226]. For example, social competence promotes child development in a number of domains, including social adjustment and interpersonal relationships [223, 227, 228]. Conversely, social competence deficits have been linked to social maladjustment and several problem behaviors, including aggression and delinquency [105, 223, 229–234].

Previous studies have primarily examined individual relationships between different types of SES, marital conflict, parenting practices, social competence, and child outcomes, without considering these associations in a comprehensive model. However, when considering the complex relationships between these variables, social competence was adversely related to later behavioral problems, as a mediating mechanism in the link between SES and child adjustment. Preschool social competence played a potential protective role in preventing later behavioral problems in the first grade. This result is an extension of previous studies, in which social competence was found to influence later adjustment, as shown in the complex relationships among these variables.

The prevailing model of prevention holds that reducing risk factors associated with adverse outcomes, and increasing protective factors that moderate the effects of exposure to risk, will reduce the possibility of later maladjustment [235]. The effectiveness of this approach towards prevention rests on the extent to which identified risk and protective factors are actually causal. Therefore, the current study findings, which focus on multidimensional family factors’ simultaneous promotion of social competence among preschoolers, may provide an effective strategy for promoting later social adjustment among children.

### Limitations and future directions

Our findings should be interpreted in light of several limitations. First, although this study’s design was longitudinal, the design was partially cross-sectional, identifying the relationship between family factors and social competence at T1. The cross-sectional design poses several restrictions that make it difficult to assume causality among the factors. Statistical evidence from studies using a cross-sectional design may not be as informative as longitudinal data [236, 237]. Prior studies have found that children’s mental health functioning influences inter-parental relationship and parenting styles, as well as the influence of inter-parental relationship and parenting styles on children’s mental health functioning [238–241]. Children’s mental health functioning and family factors are likely to influence each other. Furthermore, follow-up period of the current study was only 1 year. Although the transition period from early childhood to elementary school is an important period of mental development for children, 1 year may not be enough follow-up time to estimate the effects that have taken place, leading to the possibility of underestimating the impact of SES. Future studies should primarily focus on longitudinal research to examine the effects of family factors on later social competence. Specifically, it is necessary to have longitudinal research with surveys distributed at least three different time points and more long term to clarify the extent to which family factors flow through social competence to affect later behavioral problems.

Second, the majority of the data in this study (i.e., marital conflict, parenting practices, and child behavioral adjustment) was obtained from only mothers; therefore, there is a risk of reporting bias. This vulnerability to reporting bias can pose a serious potential problem to interpretation of the findings [242–245]. Single respondents views’ toward family factors and child mental health functioning may be skewed either more positively or negatively, thus resulting in misleading findings. The arguments for the examination of the complex relationships between components of SES, family processes, and child mental functioning would seem to be not fully realized with data provided only from mothers. Paternal and maternal education levels or other background information may also influence their views of family factors and children’s adaptive functioning; several studies have showed there are discrepancies between the views of fathers and mothers [246, 247]. Therefore, this study’s data may obscure the extent to which paternal education is associated with the inter-parental relationship, parenting styles, and children’s adaptive functioning, since information from the point of view of fathers was absent.

Furthermore, other factors may also influence the views of the informants. For example, regarding the inter-parental relationship, prior studies have shown that views of conflict vary across men and women; women tend to report more conflict episodes than men do, whether for the better or worse [248]. In addition, regarding parenting styles, the data provided by only maternal reports did not reveal information concerning fathers’ involvement. Generally, fathers and mothers each have their own parenting styles. Many studies have shown that fathers and mothers play similar or complementary roles in terms of parenting behavior, simultaneously suggesting that their qualities of parenting behavior differ, in particular concerning the amount of physical play; fathering may prove to be more challenging [249–251].

Views of children’s adaptive functioning behavior may vary across fathers, mothers, and children’s teachers. Many study findings indicate that there are several discrepancies among informants, including fathers, mothers, and children’s teachers. These discrepancies are particularly prevalent between children’s parents and teachers, in terms of their assessment of the children’s psychological well-being [242–245, 252]. The discrepancies may reflect children’s symptoms, or the opportunities to observe them. Generally, it is not easy for parents to assess early maladaptive behaviors. In particular, parents have difficulty identifying behavior that is indicative of internalizing problems in young children. For instance, it is difficult for parents to distinguish behavior that is reflective of underlying psychopathology from behavior that is reflective of immaturity in self-regulatory competence. Conversely, teachers have the advantage of having the opportunity to observe the behavior of many other children simultaneously. Furthermore, behavioral problems are likely to be more apparent at school than at home. Therefore, obtaining teacher reports may be particularly important for young children to aid in the assessment and forecasting of their school maladjustment and mental health problems [253]. Furthermore, several studies have suggested that the combination of teacher and parent reports with independent assessments is more sensitive than either assessment alone [254]. Therefore, in future studies, reports from several dissimilar informants, including those from fathers and teachers, in addition to mothers, will be needed to more precisely evaluate how family factors affect child mental health functioning.

Third, in the current study, we did not consider the interplay between maternal and paternal education, or the interplay between positive and negative aspects of inter-parental functioning. We studied the independent contributions of both maternal and paternal education, and those of the positive and negative aspects of inter-parental functioning; the framework used in this study does not lead to an examination of the actual interplay among any of these factors.

Regarding parental education, we included the independent contributions of both maternal and paternal education level, as we expected each SES indicator, as a predictor, to be differentially associated with family processes and child mental functioning through distinct pathways. However, the argument is incomplete and not generally consistent with theoretical perspectives, including family systems and developmental systems theories [1–5]. Theoretical perspectives suggest there is a more dynamic interplay than the simple additive contribution of maternal and paternal education. Not modeling the interaction between maternal and paternal education achievement may mislead the influence of each maternal and paternal education achievement.

Regarding inter-parental functioning, we also included the independent contributions of both positive and negative aspects of inter-parental functioning, as there are reasons we expected each positive and negative aspect of inter-parental functioning to be differentially associated with other variables through distinct pathways. Most studies empirically investigating the FSM have focused exclusively on the negative aspect of inter-parental functioning [15, 37]. Previous research suggests the interplay between the positive and negative aspects of inter-parental functioning is more complex than simply looking at the independent contributions of each [15, 37]. Previous research also suggests that it is not easy to distinguish the positive and negative aspects of inter-parental functioning, and that children respond to the whole instead of just the parts [29, 37–39]. The model including the independent contributions of both the positive and negative aspects of inter-parental functioning may not precisely assess the influences of each. Therefore, the inclusion of maternal and paternal education, and the positive and negative aspects of inter-parental functioning are both strengths and weaknesses of this study.

Fourth, we could not exactly assess the positive aspects of inter-parental functioning as a constructive marital conflict. As mentioned earlier, we used the Quality of Co-parental Communication (QCCS) measure to assess the positive and negative aspects of inter-parental functioning. The QCCS captures two aspects of the inter-parental relationship: Co-parental Conflict (only the negative side); and Co-parental Support (general helpfulness, resourcefulness, and cooperation) [152]. The Support subscales of this scale measured only “general support”; it has not precisely measured the constructive aspects of conflict. However, in the current study, we treated Co-parental Support, as measured by the Support subscales, as constructive conflict. Thus, the “constructive conflict” we used may not precisely assess the influence of the positive aspects of inter-parental functioning on the other variables. Future studies should investigate this possibility further by using other scales to more precisely assess the constructive aspects of conflict.

Fifth, there are likely to be other factors that were not accounted for in our model. As mentioned earlier, we found a direct association between SES and child mental health functioning, while controlling for other variables. There are likely to be other family environmental factors (e.g., child-rearing environment and more factors of the inter-parental relationship and child/parent interaction). Furthermore, although we found the effects of certain hypothesized family environmental factors on child mental health functioning, we did not consider genetic factors in our model; it is important to realize children’s behavioral problems may be influenced by genetic risks, as well as their family’s environmental factors. A large body of evidence supports the conclusion that children’s behavioral problems are moderately heritable [255–258].

Several studies have suggested the extent to which children’s mental health functioning is affected by family environmental factors depends on genetic and early temperamental characteristics; environments help determine how genes express themselves [259–261]. Children with different genetic attributes will respond differentially to the same environmental circumstances. Therefore, it is difficult to distinguish genetic effects from the effects of family environmental factors on child mental health functioning because genetic factors were not examined in this model. Consequently, there are likely to be other family environmental and genetic factors that need to be included in this model. Future studies should investigate this possibility further by including more family environmental factors related to child mental health functioning. Specifically, these studies could include a genetically informative design (e.g., a twin or adoption study design), as these types of studies would be useful in accounting for the interplay between individuals and environmental circumstances.

Furthermore, although we described earlier that the FIM contends that family SES is associated with neighborhood conditions as one aspect of parental investment, our studies did not assess areal characteristics (i.e., neighborhood conditions). Family’s socioeconomic resources are likely to largely determine the kind of neighborhood in which they reside [262]. Wealthier parents are expected to reside in areas that have a positive community environment, which provides resources for the developing child, such as parks, good schools, community involvement among residents, and access to conventional friends. Conversely, poor parents are constrained in their choice of neighborhoods. Children reared in neighborhoods without these resources experience a number of negative consequences. Lower income may lead to residing in extremely poor neighborhoods, which are characterized by few resources for child development, such as playgrounds, childcare, health care facilities, and after-school programs. Children who live in areas of disadvantaged neighborhoods tend to have poor physical and mental health [263, 264]. Furthermore, several studies suggest that the affluence of neighborhoods is associated with child outcomes over and above family poverty [265]. Thus, future studies will need to include an assessment of neighborhood quality.

Finally, these findings may not be generalizable to all families, because there is a risk of attrition bias, and the sample was drawn from a limited geographical area in an urban metropolis of Japan. As mentioned earlier, the retention rate from T1 to T2 was 51.6%, and the T2 returning participants tended to be relatively higher in SES than the non-returning participants. This indicates there is a risk of attrition bias. Therefore, there is the possibility that our analyses could not exactly evaluate the mechanism of children with lower SES, and our analyses may underestimate the influence of SES. Furthermore, some characteristics of Japanese society, such as low levels of economic disparity and high education levels among the general population, may have contributed towards the current results. The reproducibility of the current results should be confirmed using data from other regions in a variety of settings. In summary, future research on these topics would benefit from longitudinal designs and samples with higher retention rates (in particular, lower SES participants), and greater demographic and clinical diversity.

## Conclusions

Despite the above-mentioned limitations, our findings help advance our understanding of the relationships between different types of SES, marital relationships, parenting styles, and child social competence and behavioral problems. This study highlights the need to simultaneously explore the interrelations between multiple family factors to further our understanding of child mental health functioning.

Emphasis is placed on the importance of examining both family income and educational levels of parents as SES indicators, to elucidate the relationships between family factors and child adjustment. Additionally, consistent with a developmental psychopathology perspective, this study emphasizes the need to explore both positive and negative aspects of family processes (i.e., marital relationships and parenting styles), with a particular focus on the positive dimensions of family functioning. This study also emphasizes social competence as a potential protective factor that prevents later behavioral problems.

The current study advances the understanding of SES, marital conflict, and parenting, utilizing a family systems explanation for child development. (1) This study adds to previous literature concerning the relationship between SES and child mental health outcomes by demonstrating that both family income and parental education levels simultaneously and independently influence child mental health outcomes through marital conflict and parenting practices. In addition, (2) the current study adds to previous literature concerning the relationship between SES and child mental health functioning, by demonstrating the positive pathway where constructive marital conflict was shown to be related to higher levels of affirmative parenting and, in turn, more positive outcomes. The current study supports not only the notion of negative spillover effects, but also of positive spillover effects. In addition, (3) social skills, which were affected by multidimensional family factors (i.e., SES, including family income and parental education levels, and both positive and negative dimensions of family processes), adversely influenced later internalizing and externalizing behaviors. Therefore, our study suggests the possibility that theoretical models, including the FSM, should be included with parental educational levels and positive aspects of family functioning and child outcomes when examining the effects of SES.

These findings offer preliminary evidence for the need to explore SES by including family income and parental educational levels, and both negative and positive aspects of family functioning. They advance our understanding of SES, marital conflict, and parenting practices, using a family systems explanation for child development. Therefore, our results suggest that we should be sensitive to social inequalities in children’s mental health problems and developmental outcomes, and strive to reduce social inequalities. In the long-term, it may be necessary to focus not only on economic support, but also on education, as providing equal access to suitable educational opportunities can positively affect the next generation, and is likely to have a more permanent impact on the child-rearing environment than a temporary increase in income. If more parents can become better educated through an improved social system, it might lead to better developmental outcomes for children. In addition, simultaneously focusing on the marital relationship and parenting style in negative and positive domains may be an effective strategy for developing social adjustment among children. The current study suggests that marital relationships and parenting skills in negative and positive domains may be appropriate for interventions promoting social competence among children to prevent later social maladjustment among parents and children who are socioeconomically disadvantaged. Our findings have important clinical and policy implications.

## Abbreviations

QCCS: The Quality of Co-parental Communication Scale
APQ: The Alabama Parenting Questionnaire
SSQ: The Social Skills Questionnaire
SDQ: The Strengths and Difficulties Questionnaire
